# Supplementary material for: Delays in Epidemic Outbreak Control Cost Disproportionately Large Treatment Footprints to Offset
Source: Pathogens. 2022 Mar 24;11(4):393. doi: 10.3390/pathogens11040393 (PMC9030382; doi:10.3390/pathogens11040393)
Supplement: Supplementary file 1 [file pathogens-11-00393-s001.zip › pathogens-1616912-supplementary.pdf]

Table S1. Effects of ring cull size and treatment timing expressed as a percentage of the area under the disease gradient (AUDG) for untreated control epidemic. Results are for the field experiments (means) and simulations at different R0-values, outbreak disease levels, and number of latent periods.

	5 Latent Periods	5 Latent Periods				8 Latent Periods				Initial Disease Prevalence (5 Latent Periods)		
**Treatment**	**Field Experiment**	**Ro = 4**	**Ro = 20**	**Ro = 70**	**Ro =140**	**Ro = 4**	**Ro = 20**	**Ro = 70**	**Ro = 140**	**0.5%**	**5%**	**15%**
Control	100.0%	100.0%	100.0%	100.0%	100.0%	100.0%	100.0%	100.0%	100.0%	100.0%	100.0%	100.0%
1.125LP-1X	29.4%	5.5%	7.7%	9.7%	15.9%	3.5%	13.8%	40.4%	58.2%	9.8%	14.2%	21.9%
1.125LP-3X	4.1%	5.1%	7.0%	8.3%	14.6%	3.1%	12.0%	38.7%	56.4%	8.9%	13.0%	20.3%
1.125LP-5X	10.4%	4.9%	6.7%	7.6%	13.7%	2.8%	11.0%	37.8%	55.4%	8.5%	12.4%	19.3%
1.25LP-1X	40.4%	23.5%	30.0%	40.3%	53.2%	15.7%	44.0%	68.5%	83.7%	36.8%	52.6%	67.2%
1.25LP-3X	21.8%	22.0%	27.5%	35.1%	49.7%	13.7%	39.2%	66.1%	81.9%	33.7%	48.5%	63.5%
1.25LP-5X	66.1%	21.2%	26.1%	34.0%	47.5%	12.6%	36.7%	64.4%	80.9%	31.9%	45.6%	60.1%
1.5LP-1X	96.6%	49.5%	48.4%	57.6%	71.2%	34.4%	64.4%	81.6%	91.4%	54.0%	73.7%	88.3%
1.5LP-3X	117.1%	46.7%	44.7%	54.2%	67.1%	30.2%	59.3%	79.0%	90.0%	49.5%	68.5%	84.3%
1.5LP-5X	95.7%	45.2%	42.6%	50.7%	64.1%	27.8%	56.1%	77.4%	89.0%	47.0%	65.4%	82.0%

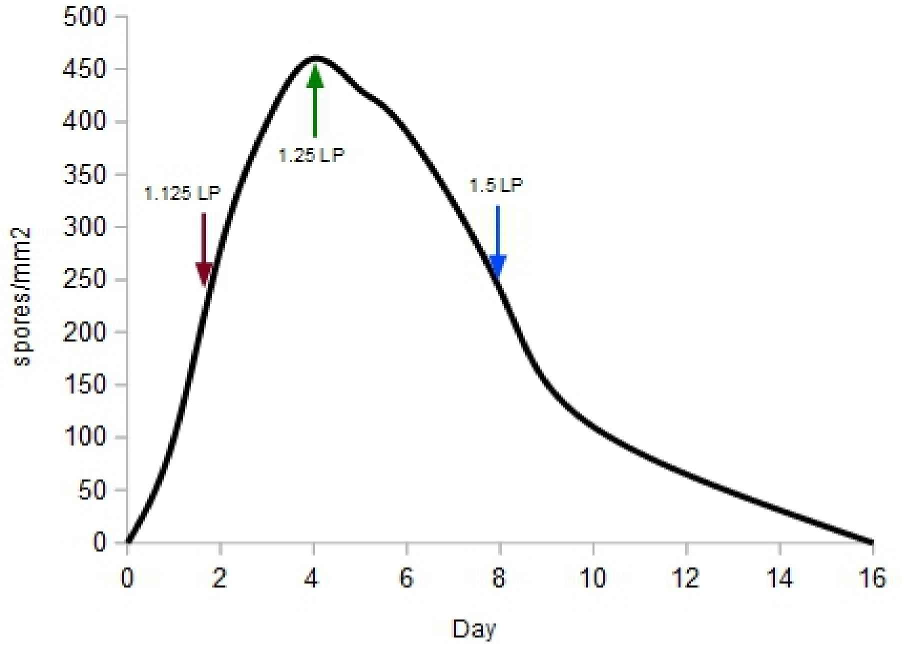

Figure S1. Spore production curve (Papastamati and van den Bosch [68], Fig. 1D, year 2006) used to estimate the proportion of the total number of spores released by each timing treatment. Arrows indicate the approximate positions of each ring cull timing treatment. 1.125 LP (Latent Periods) = a red arrow, 1.25 LP = a green arrow, 1.5 LP = a blue arrow.

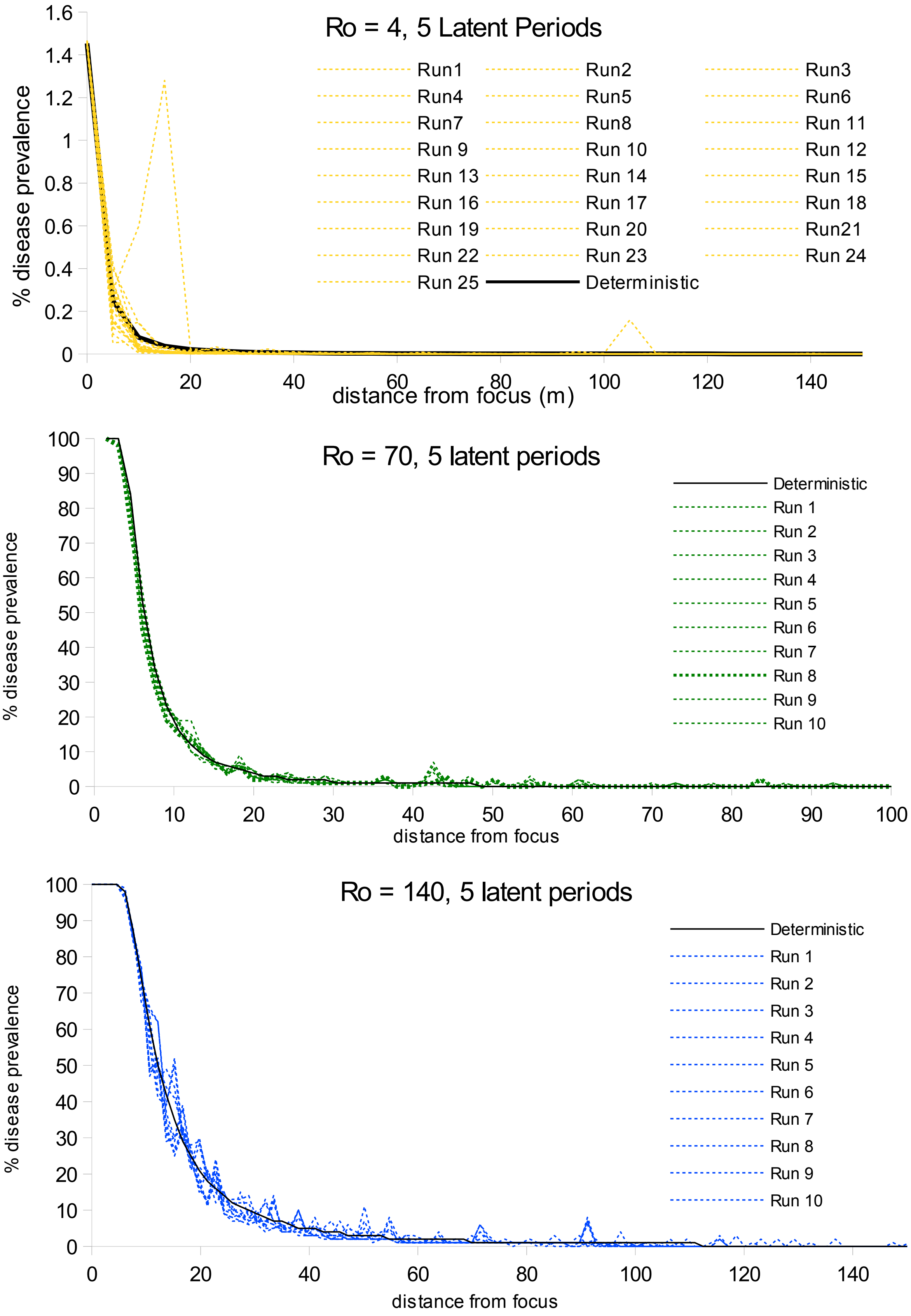

Figure S2. Random selection of disease gradients, produced by 300 stochastic (dispersal distance) runs (colored lines) at different R0-values holding outbreak disease prevalence at 1.2% and one deterministic run (black line), at the end of 5 latent periods. The effects of ring cull size and treatment timings were in most instances greater than the variation due to dispersal stochasticity, so we opted to present only the results from deterministic runs.

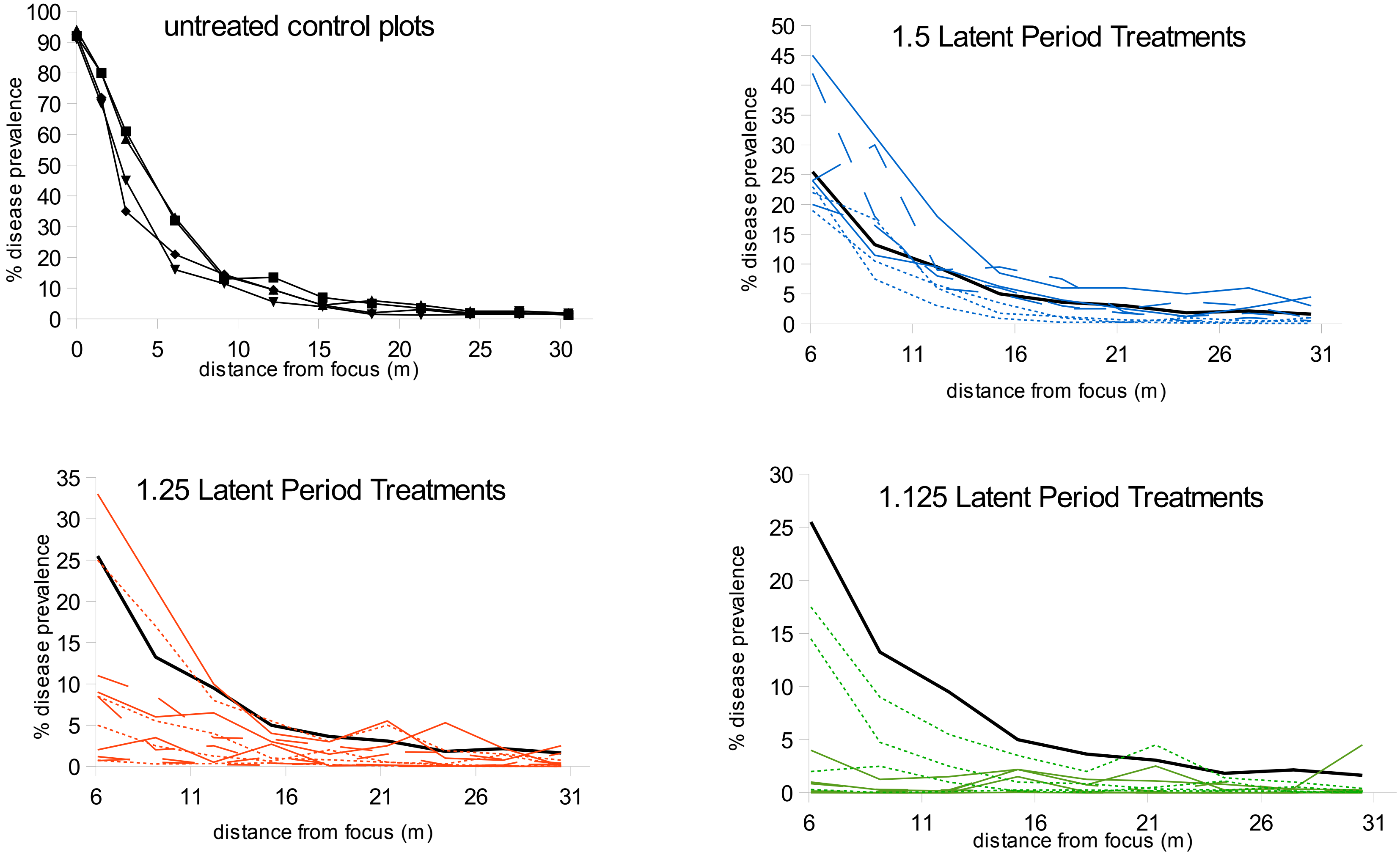

Figure S3. Wheat stripe rust disease gradients from individual plots in the field experiment, arranged by treatment timing 1.125 LP (latent periods following disease initiation), 1.25 LP, 1.5 LP, and an untreated control. For all figures except the control plots: dotted lines represent 1X ring cull size, hashed lines represent the 3X ring cull size, solid colored lines represent the 5X ring cull size, and the solid black line represents the mean of the control disease gradients.
